# Self-reported arterial hypertension, use of health services and guidelines for care in Brazilian population: National Health Survey, 2019

**DOI:** 10.1590/SS2237-9622202200012.especial

**Published:** 2022-08-08

**Authors:** Deborah Carvalho Malta, Regina Tomie Ivata Bernal, Elton Junio Sady Prates, Nádia Machado de Vasconcelos, Crizian Saar Gomes, Sheila Rizzato Stopa, Luciana Monteiro Vasconcelos Sardinha, Cimar Azeredo Pereira

**Affiliations:** 1 Universidade Federal de Minas Gerais, Departamento de Enfermagem Materno Infantil e Saúde Pública, Belo Horizonte, MG, Brazil; 2 Universidade Federal de Minas Gerais, Programa de Pós-Graduação em Enfermagem, Belo Horizonte, MG, Brazil; 3 Universidade Federal de Minas Gerais, Escola de Enfermagem. Belo Horizonte, MG, Brazil; 4 Universidade Federal de Minas Gerais, Programa de Pós-Graduação em Saúde Pública, Belo Horizonte, MG, Brazil; 5 Ministério da Saúde, Departamento de Análise de Saúde e Vigilância de Doenças não Transmissíveis, Brasília, DF, Brazil; 6 Instituto Brasileiro de Geografia e Estatística, Diretoria de Pesquisas, Rio de Janeiro, RJ, Brazil

**Keywords:** Hypertension, Primary Health Care, Health Services Accessibility, Brazil, Cross-Sectional Studies, Health Surveys

## Abstract

**Objective:**

To describe the prevalence of arterial hypertension according to sociodemographic characteristics in Brazil and to analyze the indicators related to access to health services and guidelines for controlling the disease in the country.

**Methods:**

Cross-sectional descriptive study using the National Health Survey (PNS) conducted in 2019. The prevalence of hypertension was estimated with a 95% confidence interval, in addition to the proportions of hypertension indicators.

**Results:**

There were 88,531 respondents, of which 23.9% self-reported hypertension, more prevalent among females (26.4%) and the elderly (55.0%). Among those who self-reported hypertension, 57.8% reported medical attention in the last six months; most received guidance on self-care; 66.1% were seen in public health services; and 45.8%, in primary health care units.

**Conclusion:**

The prevalence of hypertension in the Brazilian population was high, with most people who self-reported the condition being seen in services of the Brazilian National Health System (SUS), where they received guidance on health promotion.

**Study contributions**

**Main results**

The prevalence of self-reported arterial hypertension was 23.9%, more prevalent among females and the elderly. Most received guidance on self-care; 66.1% were treated by the public health service and 45.8% at a primary health care center.

**Implications for services**

Most participants’ last appointment was at a primary health care center, which is, therefore, a gateway to the care of people with hypertension, and essential for accessing health promotion initiatives, medicines, exams and referrals to specialists.

**Perspectives**

Health care is essential for the reduction of modifiable risk factors that impact the occurrence of hypertension. The continuous assessment of access to health helps in the development of public policies that improve the population's quality of life.

## Introduction

Arterial hypertension is defined based on persistently high blood pressure (BP) measurements, with systolic BP (SBP) greater than or equal to 140 mmHg and/or diastolic BP (DBP) greater than or equal to 90 mmHg.^1^ It is well known that hypertension is characterized as a multifactorial condition, resulting from genetic/epigenetic, environmental, social and cultural factors, and lifestyle.^1^

Worldwide, it is estimated that arterial hypertension is responsible for 10.4 million annual deaths and 218 million disability-adjusted life years (DALYs)^2^ in addition to being the attributable cause of approximately 40% of deaths in persons with diabetes *mellitus*, 14% of maternal-fetal mortality during pregnancy and 14.7% of total DALYs for chronic kidney disease.^2^ ^,^ ^3^ In Brazil, previous studies^4^ ^,^ ^5^ have shown an increasing trend in the prevalence of hypertension among the adult population, and the Chronic Disease Risk and Protective Factors Surveillance Telephone Survey (VIGITEL) found that, in 2018, 24.7% of the respondents self-reported such a diagnosis.^6^

Hypertension is one of the main modifiable risk factors for cardiovascular disease, chronic kidney disease, glucose intolerance, diabetes *mellitus*, dyslipidemia and abdominal obesity,^1^ ^,^ ^7^ ^,^ ^8^ besides having a great impact on medical and socioeconomic costs derived from complications in the target organs.^1^ ^,^ ^9^ In 2018, for example, the direct costs of the Brazilian National Health System (SUS) with hypertension were estimated at more than 2 billion BRL.^9^

Hypertension prevention is cost-effective and evidence demonstrates the relevance of primary and secondary prevention measures, with actions for the prevention, early detection and control of hypertension in Primary Health Care (PHC) programs.^10^ PHC plays a key role in health promotion, in the democratization of universal access to health services, and in acting directly in the improvement of health indicators and the reduction of potential years of life lost.^11^ Health education and health promotion actions, in turn, help the individuals in terms of improved understanding of their needs and aspirations and, thus, enabling them to take greater control over their well-being.^12^ Consequently, care for individuals with hypertension proves to be crucial to increase the quality of life and mitigate the social and economic impacts of this condition on families, governments and health systems.

In view of the above, this study aimed to describe the prevalence of hypertension according to sociodemographic characteristics in Brazil and to analyze the indicators related to access to health services and guidelines for the control of the disease in the country.

## Methods

This was a descriptive cross-sectional study that analyzed data from the National Health Survey (PNS) conducted in 2019 by the Brazilian Institute of Geography and Statistics (IBGE) in partnership with the Ministry of Health.

In order to calculate the sample size of the PNS, the following aspects were considered: the mean values, the variances, and the effects of the sampling plan, and a non-response rate of 20% was assumed. The sampling methodology is better detailed in a specific publication.^13^ The PNS sampling consisted of a cluster plan, with three stages of selection: census sectors or set of sectors (primary units), households (secondary units) and adult residents (tertiary units). In 2019, in the third selection stage, a resident aged ≥ 15 years was randomly selected, based on a list previously obtained.^13^ For the analysis of the present study, only residents aged ≥ 18 years who were selected for the interview were considered.

Regarding the variables, questions from the Q module on chronic non-communicable diseases (NCDs) referring to the topic of hypertension were used. The outcome variable was self-reported hypertension, defined as a positive answer to the question: *Has a doctor ever diagnosed you with hypertension (high blood pressure)?* (yes; no).

The sociodemographic characteristics analyzed were: sex (male; female), age (by age group: 18-24; 25-39; 40-59; ≥ 60 years old), education (no schooling/incomplete elementary school; complete elementary education/incomplete high school; complete high school/incomplete higher education; complete higher education) and self-reported race/skin color (White; Black; Brown). Despite the fact that individuals with Yellow and Indigenous race/skin color are included in the total, the analysis of their data was not carried out discriminatively, according to recommendations from the IBGE, due to the small number of observations and high coefficient of variation.

Subsequently, among those who self-reported a diagnosis of hypertension, the proportions of the variables derived from the questions were analyzed, as described below:

- Care for hypertension: *Do you go to the doctor/health care service regularly for monitoring of hypertension (high blood pressure)?* (yes; no; never); *Last visit?* (less than 6 months ago; 6 months ago to less than 1 year ago; 1 year ago to less than 2 years ago; 2 years ago to less than 3 years ago; 3 years ago or more; never); *Place of last care?* (primary health care center; private practice office; emergency care center; public hospital outpatient clinic; public polyclinic; private emergency care center; home; pharmacy; other services); *Was this health care provided by SUS?* (yes; no; don't know).
- Payment: *Did you pay any amount for this care/service?* (yes; no).
- Pharmacological treatment: *Has any doctor ever prescribed you any medication for high blood pressure?* (yes; no); *In the past two weeks, have you taken medication to control hypertension (high blood pressure)?* (yes, all of them; yes, some; no).
- Guidelines for hypertension: *In any of these consultations for hypertension, did a doctor or another health professional give you any of these recommendations?* (yes; no, the recommendations being: maintain a healthy diet, maintain adequate weight, ingest less salt, practice regular physical activity, do not smoke, do not drink excessively, follow up regularly with a health professional and indication of integrative practices).
- Request for tests: *In any of these consultations for arterial hypertension, was a test requested?* (yes; no, the possible tests being: blood, urine, electrocardiogram or stress test).
- Referral to a specialist: *In any of the consultations for arterial hypertension, was there a referral to a specialist, such as a cardiologist or nephrologist?* (yes; no).

The original PNS questionnaire is available on the following website (https://www.pns.icict.fiocruz.br/questionarios/), where more details on the categories of the variables can be found. Data collection took place between August 2019 and March 2020. For the present study, the database released by IBGE was extracted, through the electronic address https://bit.ly/3Kp5Q8c, and analyzed in November 2020.

For the statistical analysis, the variables were transformed into dichotomous variables, with the answer “yes” being equal to “1”, and the others equal to “0”. Prevalence and age-adjusted prevalence ratios were calculated with their respective 95% confidence intervals (95%CI). Subsequently, Pearson's chi-square test was applied to compare the prevalence of total hypertension and by sex, according to sociodemographic characteristics, at a significance level of 5%. For the variables related to access to health services and care, the percentage was calculated among respondents who self-reported a diagnosis of hypertension, for each category, with their respective 95%CI.

Due to the complex sampling design and unequal selection probabilities, the PNS analysis requires sample weights for selected households and residents.^13^ The final weighting used is the product of the inverse of the selection probability expressions at each stage of the sampling plan and includes the correction for non-responses and adjustments to total populations.^13^ The Statistics and Data Science software (StataCorp LP, CollegeStation, Texas, United States) version 14.0 was used for data analysis through the survey module, which considers the effects of the sampling plan.

The consent from all participants was obtained directly on the device at the moment of the interview. The PNS project was sent to the National Committee for Ethics in Research/National Health Council and approved under No. 3,529,376, issued on August 23, 2019. The present study used secondary, unidentified and public domain data from the PNS and, therefore, there was no need for further assessment by the Committee for Ethics in Research.

## Results

**Figure 1:**
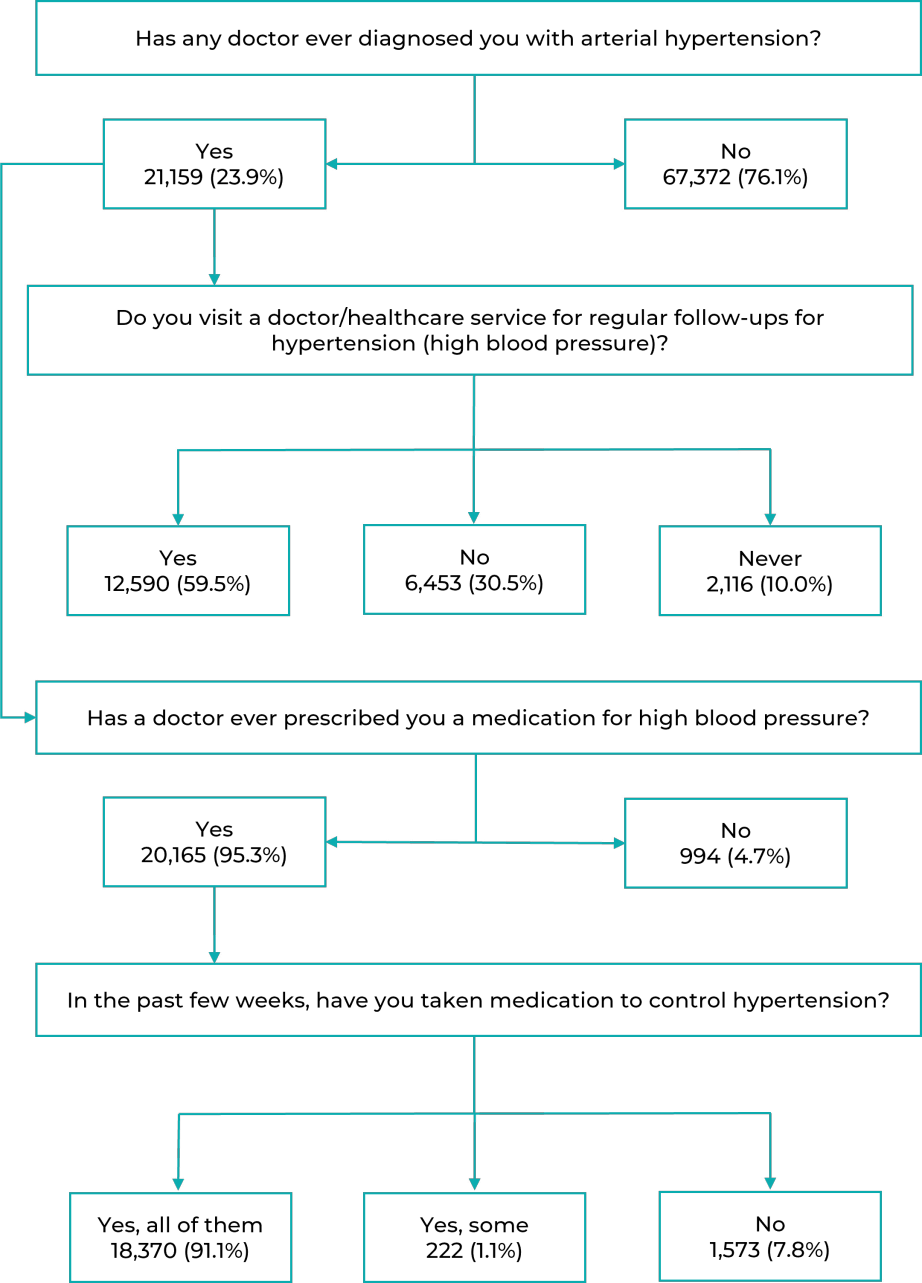
Flowchart of the distribution of study participants (n = 88,531) according to the self-reported hypertension questionnaire, National Health Survey, Brazil, 2019

The sample planned for the 2019 PNS was 108,525 households and data were collected from 94,114 (a response proportion of 86.7%). In this study, data from 88,531 individuals were analyzed. Figure 1 shows the flowchart of questions referring to the self-reported diagnosis of hypertension, showing the number of respondents and the flows followed in the questionnaire.

Among adults who reported hypertension only 59.5% reported going to the doctor/health service regularly to monitor hypertension; 30.5% reported not going to the doctor; and 10.0% never sought services regularly for hypertension control. Also, 95.3% stated that they had been prescribed some type of medication for hypertension and, among these, 91.1% had taken all the medications in the past two weeks (Figure 1).

The prevalence of self-reported hypertension, according to previous medical diagnosis, was 23.9% (95%CI 23.5;24.4), being higher among females (26.4%; 95%CI 25.8;27.1). People aged ≥ 60 year (55.0%; 95%CI 53.9;56.1) had a 22 times higher prevalence of hypertension, compared to those aged between 18 and 24 years (2.3%; 95%CI 1.7;2.9). The population with higher education (18.2%; 95%CI 17.1;19.3) had a 30% lower prevalence compared to those with the lowest education (36.6%; 95%CI 35.7;37.4). In relation to self-reported race/skin color, Black people (25.8%; 95%CI 24.4;27.2) and White people (24.4%; 95%CI 23.6;25.2) showed a higher prevalence, when compared with individuals of Brown race/skin color (22.9%; 95%CI 22.2;23.6). In the analysis by sex, the prevalence remained higher among females for people aged ≥ 60, less educated and in all self-reported race/skin color (Table 1).

**Table 1:** Prevalence and adjusted prevalence ratio of arterial hypertension according to sociodemographic characteristics, National Health Survey, Brazil, 2019

Variables	Total		Female		Male	
	Prevalence (95%CI)^a^	PR^b^	Prevalence (95%CI)^a^	PR^b^	Prevalence (95%CI)^a^	PR^b^
**Total**	23.9 (23.5;24.4)	26.4 (25.7;27.2)	21.1 (20.4;21.8)
**Age group (years)**						
18-24	2.3 (1.7;2.9)	1.0	2.1 (1.5;2.8)	1.0	2.5 (1.6;3.4)	1.0
25-39	7.3 (6.7;7.8)	3.2	7.2 (6.3;8.0)	3.5	7.4 (6.6;8.1)	3.0
40-59	27.2 (26.3;28.1)	11.5	29.5 (28.2;30.7)	13.3	24.6 (23.3;25.9)	9.9
≥ 60	55.0 (53.9;56.1)	22.1	59.4 (57.9;60.8)	24.7	49.3 (47.6;50.9)	19.7
**Education**						
No schooling/Incomplete elementary school	36.6 (35.7;37.5)	1.0	43.3 (42.0;44.6)	1.0	29.2 (28.0;30.3)	1.0
Complete elementary school/Incomplete high school	20.4 (19.1;21.6)	1.0	24.7 (22.8;26.6)	0.9	16.2 (14.6;17.8)	1.0
Complete high school/Incomplete higher education	15.4 (14.7;16.2)	0.8	15.7 (14.7;16.7)	0.8	15.1 (14.0;16.2)	1.0
Complete higher education	18.2 (17.1;19.3)	0.7	16.3 (15.0;17.7)	0.6	20.7 (18.8;22.6)	1.0
**Self-reported race/skin color**						
White	24.4 (23.6;25.2)	1.0	26.0 (24.9;27.1)	1.0	22.5 (21.4;23.5)	1.0
Black	25.8 (24.4;27.2)	1.2	30.2 (28.2;32.2)	1.2	20.9 (19.1;22.7)	1.1
Brown	22.9 (22.2;23.6)	1.1	25.7 (24.8;26.7)	1.1	19.7 (18.8;20.6)	1.0

a) 95%CI: 95% confidence intervals; b) PR: Adjusted prevalence ratio.

Regarding medical care due to hypertension, 72.2% of the respondents reported having received medical care in the last 12 months and, of these, 57.8% in less than 6 months before the interview. Medical care for hypertension occurred mainly in public health services, totaling 66.1%, 45.8% of which took place at the PHC center. Most participants reported having received guidance on self-care for hypertension, such as: maintaining a healthy diet (87.2%), maintaining adequate weight (84.3%), ingesting less salt (87.7%), engaging in regular physical activity (PA) (81.7%), not smoking (67.2%), and not drinking excessively (66.5%), in addition to regular follow-up with a health professional (85.2%). Among the requested tests, it was observed that a blood test was requested in 79.9% of the cases, a urine test in 69.9%, an electrocardiogram in 64.5% of the cases and a stress test in 33.6%. 25.0% of the individuals with hypertension were referred to specialists for an appointment (Table 2).

**Table 2:** Percentage distribution and 95% confidence intervals of care-related characteristics of people who self-reported hypertension (n = 38,082), National Health Survey, Brazil, 2019

Variables	%	95%CI^a^
**Last visit to healthcare service**		
Less than 6 months ago	57.8	56.6;59.0
From 6 months ago to less than 1 year ago	14.4	13.6;15.2
From 1 year ago to less than 2 years ago	9.4	8.8;10.1
From 2 years ago to less than 3 years ago	2.7	2.3;3.2
3 years ago or over	13.7	12.9;14.5
Never	1.9	1.6;2.3
**Location of the last healthcare visit**		
Primary health care center	45.8	44.4;47.2
Private doctor’s office	28.8	27.5;30.1
Emergency care center	9.6	8.9;10.5
Public hospital outpatient clinic	7.1	6.5;7.8
Public polyclinic	3.6	3.1;4.2
Private emergency care center	1.7	1.4;2.0
At home	1.4	1.2;1.6
Pharmacy	0.9	0.7;1.2
Other services	1.0	0.7;1.4
Guidelines		
Integrative practices	7.4	6.8;8.1
Not drinking excessively	66.5	65.1;67.8
Not smoking	67.2	65.8;68.6
Engaging in regular physical activity	81.7	80.7;82.7
Maintaining adequate weight	84.4	83.4;85.4
Regular follow-ups	85.2	84.2;86.2
Healthy eating	87.2	86.3;88.2
Reducing salt intake	87.8	86.7;88.8
**Exams and referrals**		
Blood test	79.9	78.8;80.9
Urine test	69.9	68.5;71.2
Electrocardiogram	64.5	63.2;65.8
Exercise test	33.6	32.2;34.9
Referral to see a specialist	25.0	23.7;26.2

a) 95%CI: 95% confidence intervals.

**Figure 2:**
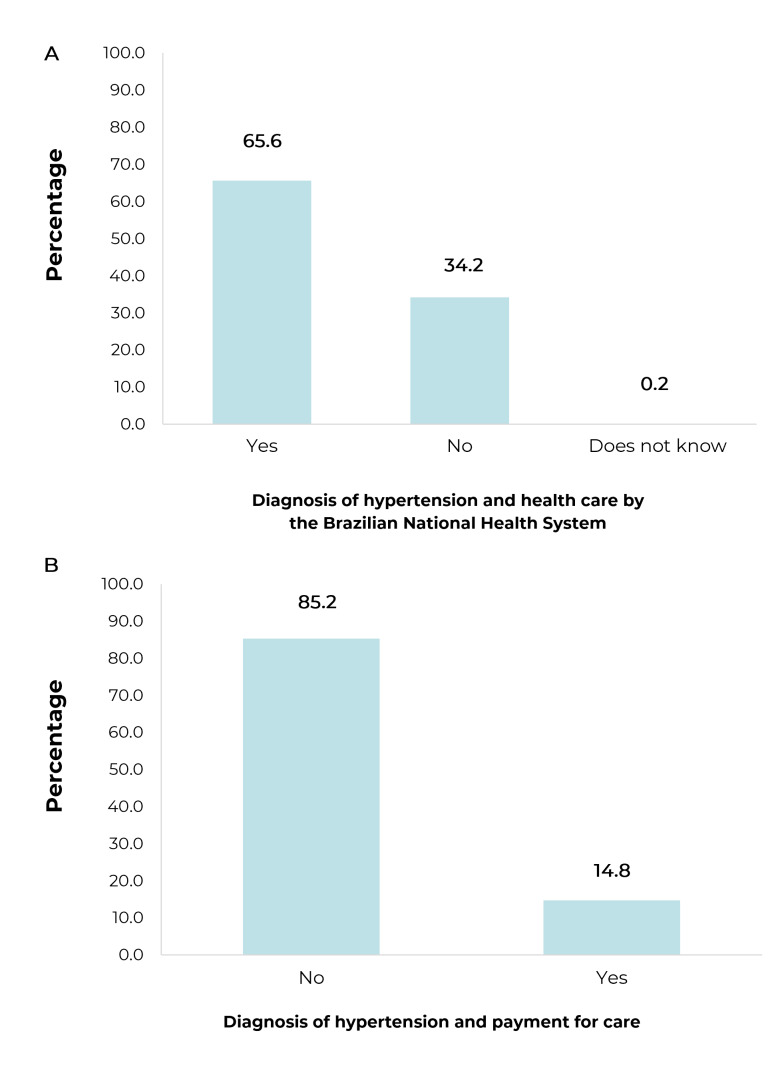
Percentage distribution and 95% confidence interval of adults (n = 38,082) who reported a diagnosis of hypertension and health care by the Brazilian National Health System (A) and payment for care (B), National Health Survey, Brazil, 2019

Lastly, 65.6% of the respondents who self-reported hypertension reported care by the SUS and only 14.8% reported payment for care related to hypertension (Figure 2).

## Discussion

The study identified that self-reported hypertension was reported by about 1/4 of the population and had higher prevalence in females, older people and among the population with low education. As for the care provided, it was found that 3/4 of the total assistance was provided by the SUS and there was extensive guidance on health promotion, which suggests that the 2020 Brazilian Guidelines on Hypertension are being systematically applied in PHC.

The PNS is the broadest and most important health survey carried out in the country. It takes place every five years^13^ and enables not only monitoring the risk and protective factors for NCDs, including self-reported hypertension, but also assessing the population's access to health services and the quality of care provided.

Although higher prevalence of hypertension was observed among females, measured BP data from the 2013 PNS showed higher prevalence among males when compared to females (25.8% and 20%, respectively).^4^ This finding can be explained by the fact that hypertension was estimated using self-reported data and females seek health services more often and, therefore, have a greater opportunity for diagnosis, besides showing greater consistency in self-care reports.^14^

The increase in the prevalence of hypertension with advancing age, consistent with the literature, leads to a significant increase in expenditure by the health system, resulting from the increase in demand. There is evidence that hypertension is associated with ageing due to lower arterial compliance and progressive stiffening of the great arteries.^1^ ^,^ ^15^

Arterial hypertension was higher in people with low education, which can be explained by the greater exposure to risk factors and adverse socioeconomic conditions, such as: lack of access to health services and lower access to guidelines on healthy lifestyles, as well as fewer opportunities of access to healthy food, physical activity and health care.^16^

The present study found a lower prevalence among individuals of Brown race/skin color and higher among Black and White individuals. There is divergence in the literature as to whether higher prevalence of hypertension in individuals with self-reported Black race/skin color could be due to genetic predisposition, in addition to determinants such as socioeconomic conditions, stress due to exposure to racism or lifestyles.^15^ As the present study is descriptive and cross-sectional, the differences found need to be explored in future analyses.

In the context of PHC center, care for individuals with hypertension must be person-centered and with an emphasis on health promotion, increasing access to information about health, in a horizontal and understandable way, seeking to assist in the decision of self-care, through medical appointments, home visits and educational activities in groups.^17^ ^,^ ^18^ There is also evidence that BP is better controlled if addressed by a multiprofessional team and, especially, the most recent edition of the Brazilian Guidelines on Arterial Hypertension^1^ has established the relevance and scope of the team's line of action in the context of PHC.

Comprehensive and multidisciplinary care is essential for reducing morbidity and mortality and, above all, for health promotion aimed at adults with hypertension. Within the multidisciplinary team, the physician is responsible for the diagnosis and risk stratification, in addition to evaluating the therapeutic regimen, whether pharmacological or not, every 6 months. Nurses should encourage self-care, promote educational actions and help the user understand and create routines and habits that develop adherence to the prescribed behaviors. Physical education professionals are committed to promoting healthy habits, reducing sedentary lifestyles and encouraging the community to become physically active and thus maintain a better quality of life. In addition, the nutritionist should advise higher consumption of vegetables and fruits, and reduced sodium intake, with the objective of maintaining weight within the normal range.^1^

In the present study, it was found that most of the prevention and treatment protocols have been followed by health professionals. Weight management guidelines were given in almost 90% of cases. It is intended, through this guideline, to promote the achievement of the ideal weight. The importance of body adiposity, especially visceral adiposity, as a risk factor for BP elevation, has already been demonstrated, as well as the fact that it may be directly connected with up to 75% of hypertension cases.^19^

It was observed that the guidelines on healthy eating were highly disseminated, as well as the guidelines for reducing sodium intake. The ideal diet should contain a daily intake of less than 2 g of sodium or 5 g of salt..^20^ A meta-analysis carried out in China in 2019 showed that intervention studies that replace sodium chloride with potassium chloride achieve a significant reduction in both SBP (-5.7 mmHg; 95%CI -8.5;-2.8) and DBP (-2.0 mmHg; 95%CI -3.5;-0.4).^21^ There is evidence that a diet rich in fruits, vegetables, grains and low fat can result in a 2/3 mmHg reduction in BP measurement. Likewise, a reduction in saturated and trans fat can reduce BP by 3 mmHg, while increasing dietary potassium intake by 3.5 to 5.0 g/day can result in a 2 mmHg reduction in BP.^1^ Studies also suggest that adherence to a diet with a higher content of fruits and vegetables is associated with a lower risk of stroke,^22^ cardiovascular mortality and chronic kidney disease.^23^

Recommendations on PA were given to more than 80% of adults with hypertension. The decrease in sedentary time reduces the risk of all-cause mortality^24^ and, associated with regular PA (150 min/week), with the incentive of aerobic PA, reduces the incidence of hypertension^25^ and can reduce BP by 5/7 mmHg.^1^

Guidelines on alcohol and tobacco reduction were less prescribed, around 60%. Reducing the consumption of alcoholic beverages is associated with a drop of about 5.5 mmHg (95%CI 6.70;4.30) in SBP and 3.97 (95%CI 4.70;3.25) in DBP.^1^ ^,^ ^26^ Tobacco use raises BP by approximately 5 to 10 mmHg.^27^ Due to the increase in cardiovascular risk, the 2020 Brazilian Guidelines on Arterial Hypertension emphasize the importance of smoking cessation, including by means of pharmacological therapy.^1^

In order to support a complete treatment, identification of the clinical and subclinical lesions of the target organs is essential. To this end, some simple and low-cost tests must be requested at the first medical appointment and then annually for all individuals diagnosed with hypertension.^1^ The following are recommended: serum potassium, serum uric acid, creatinine, blood glucose and lipid profile; proteinuria/albuminuria and urinalysis and electrocardiogram for possible detection of left ventricular hypertrophy.^1^ Some populations may need other tests, specific to their characteristics.

Most participants’ last medical appointment took place at an PHC center and, in addition to emergency care centers, almost 2/3 of them received care in locations under the public health system. Therefore, this study corroborates the organizing and guiding role of the SUS, which is the gateway and place of care and follow-up for the majority of the Brazilian population.^28^ Furthermore, the PHC center provide guidelines for the care of people with hypertension, from guidelines for health promotion actions (tobacco prevention, alcohol consumption reduction, healthy eating and PA), as well as free access to medicines, tests, exams and referrals to specialists.

The prevalence of hypertension found in the present study was lower than the global prevalence indicated in 2010 by the Global Burden of Diseases (31.0%),^29^ but it was similar to that found in previous studies in the Brazilian population. A population-based study conducted in the city of São Paulo showed, in 2015, a prevalence of hypertension of 23.2% in adults and 54.9% in the elderly.^16^ Also consistent with the present study are the percentages found in a study that used data from the 2013 VIGITEL, which were 24.1% for adults and 60.4% for the elderly aged ≥ 65 years.^15^ A study carried out using data from the 2013 PNS, in which hypertension was defined using BP measurements taken during the interview, showed a prevalence of 22.8% in adults and a lower percentage (47.1%) among the elderly over age 75.^4^

Among the limitations of the study, its cross-sectional design stands out, due to the fact that it does not allow determining causality. In addition, greater access to health services and information by the participants can be pointed out with bias when using self-reported diagnoses. Finally, the quality of care was not evaluated, and the possible differences in terms of access to health services, between the population that uses complementary health care and those who are exclusive users of the SUS, were not explored.

In conclusion, hypertension presents a high burden of morbidity and mortality in Brazil, being the main risk factor for death (17% of the total) and the second in the ranking of DALYs (responsible for 8.33% of the total) in 2019.^3^ In light of this, health care is an important step towards reducing modifiable risk factors (tobacco consumption, physical inactivity, harmful use of alcohol and unhealthy diets) that impact the occurrence of NCDs and the global burden of diseases, thus helping to ensure good health and the well-being of the population, topics included in the 2030 Agenda for Sustainable Development. Therefore, it is essential to evaluate the access to health and the satisfaction of the users of the system with the care they receive, as such information plays a crucial role in the development of evidence-based public policies aimed at improving the health and quality of life of Brazilians.
